# Attention Recurrent Neural Network-Based Severity Estimation Method for Early-Stage Fault Diagnosis in Robot Harness Cable

**DOI:** 10.3390/s23115299

**Published:** 2023-06-02

**Authors:** Heonkook Kim, Hojin Lee, Seongyun Kim, Sang Woo Kim

**Affiliations:** Department of Electrical Engineering, Pohang University of Science and Technology, Pohang 37673, Republic of Korea; kimhk85@postech.ac.kr (H.K.); suvvus@postech.edu (H.L.); ksy3dmbe3kor@postech.ac.kr (S.K.)

**Keywords:** soft fault, condition monitoring, machine learning, anomaly detection, fault estimation

## Abstract

Cable is crucial to the control and instrumentation of machines and facilities. Therefore, early diagnosis of cable faults is the most effective approach to prevent system downtime and maximize productivity. We focused on a “soft fault state”, which is a transient state that eventually becomes a permanent fault —open-circuit and short-circuit. However, the issue of soft fault diagnosis has not been considered enough in previous research, which could not provide crucial information, such as fault severity, to support maintenance. In this study, we focused on solving soft fault problem by estimating fault severity to diagnose early-stage faults. The proposed diagnosis method comprised a novelty detection and severity estimation network. The novelty detection part is specially designed to deal with varying operating conditions of industrial applications. First, an autoencoder calculates anomaly scores to detect faults using three-phase currents. If a fault is detected, a fault severity estimation network, wherein long short-term memory and attention mechanisms are integrated, estimates the fault severity based on the time-dependent information of the input. Accordingly, no additional equipment, such as voltage sensors and signal generators, is required. The conducted experiments demonstrated that the proposed method successfully distinguishes seven different soft fault degrees.

## 1. Introduction

Cable is widely utilized because it is a crucial part of control and instrumentation in industrial fields, including power plants [1,2], vehicles [3], ship building [4], and factory automation [5]. To maintain the stability and reliability of the system operations, timely diagnosis of cable fault is vital. In particular, the early diagnosis of faults can ensure the maximization of productivity by reducing unwanted system downtime in fully automated processes. A single fault in an automated process can stop the related processes because the process controllers are interlocked with each other to prevent harmful effects to other facilities and dangerous hazards to workers when a fault is detected.

There are two states of cable faults: transient and permanent. The latter represents a permanent fault that results in the failure of machines and facilities without exception. This fault consists of open and short circuits and is referred to as a hard fault. The former is a transient state, which develops according to the external conditions, thereby resulting in the accumulation of small modifications in the cable. Because of harsh operating environments, the components of the cable, which include insulators, shields, and conductors, may experience partial damage. Therefore, this is referred to as a soft fault [6]. In reality, a soft fault is not an acute fault state and does not cause significant problems to machines and facilities at that moment. However, there is a high probability of it transforming into a hard fault, which deteriorates the operation of machines and facilities, i.e., causes unwanted system outage. In practical cases, the accumulation of damage to a limit close to a hard fault can yield intermittent symptoms or show no symptoms. The case of no symptoms is problematic because the latent faults cannot be detected through system diagnosis or identified by the maintenance staff. Thus, subsequent partial damage can accumulate in the cable if there is no maintenance. Consequently, soft faults often become hard faults. Therefore, early and accurate diagnosis of soft faults before they become uncontrollable is the most effective approach to deal with practical cable problems in the field.

Traditional cable diagnosis methods are largely categorized into electrical methods (partial discharge), chemical methods (oxidation induction time), and mechanical methods (elongation at break). Despite its wide usage in practice, these methods have limited applicability in on-site field applications when the machine is working because the online diagnostic ability is lacking or too sensitive to operating noise [1]. Moreover, access to the installed cable is largely limited in automated factories; thus, applying traditional techniques is not preferred in field conditions.

Recently, machine learning-based methods, including convolutional neural networks (CNN) and recurrent neural networks (RNN), have been studied in relation to fault diagnosis. These methods can automatically extract features from the input signal, which overcomes the problem of manual feature extraction requiring domain knowledge and being time consuming, particularly when a large amount of data are to be processed. Machine learning-based diagnosis methods can be categorized into two types: those that utilize the reference signal, which is to be injected into cables, and those that utilize the output signals from operating cables, such as the current signals flowing through the cable. Many studies have reported remarkable results in diagnosing faults using a reference signal. A regression-based multilayer perceptron method with clustering algorithms was developed to detect and classify hard faults of a multicore cable by analyzing the reflected signal of the reference signal in [7]. In [8], the reflected signals were converted into combined time-frequency images using Wigner–Ville distribution. Subsequently, the images were classified using a convolutional neural network (CNN). A 1D-CNN based diagnosis method was applied to diagnose soft faults in control cables of electric motors [9]. Although methods using reference signals have shown remarkable results, the reflected signal compensation and environment noise cancelation must be conducted prior to the diagnosis of faults. Additionally, there are physical limitations related to the irregular responsibility of propagating reference signals when they encounter fault locations [6]. Furthermore, additional equipment must be prepared to conduct the diagnosis owing to the requirement of a signal-injecting device and a signal generator. Several studies have used signals from operating cables to diagnose faults. In [10], a fast fault-detection method for a power transmission line was developed using the summation of the squared three-phase currents. In [11], a novelty detection-based diagnosis method was introduced using an adversarial autoencoder and an anomaly scoring technique with three-phase current signals. The method is based on the concept that the data samples with high anomaly scores can be considered as faults [12,13,14,15,16,17,18]. The utilization of current signals for diagnosing faults is relatively reliable under environmental noise while also being cost effective, as the need to install additional equipment is eliminated. Although these methods have exhibited high accuracy in diagnosing cables, studies that focus on diagnosing the early stage of a fault—that is, a soft fault—are scarce. Moreover, fault severity estimation has not been studied in this regard. Such estimation results can provide informative guidelines when considering cable maintenance strategies in the field.

In the present study, we propose a soft fault diagnosis method that detects the presence of soft faults and estimates the fault severity in a cable using an autoencoder and an attention-based RNN. To the best of our knowledge, this is the first study to attempt to estimate soft fault severity in practical cable application using only current signals without any special equipment. First, the autoencoder network detects the novelty and calculates the anomaly score of the time-series input from the current signals. Subsequently, we construct an estimation algorithm based on a long short-term memory (LSTM) network to deal with time series data [19]. The estimation network comprises an encoder and a decoder. The decoder focuses on certain hidden states that significantly influence the output by adopting the attention mechanism (AM) [20]. The two networks for novelty detection and fault severity estimation are connected serially. The novelty detection network receives the zero-sequence current (ZSC) and the squared current ratio (SCR) as inputs, and subsequently outputs the anomaly score that directly represents the novelty of the fault, whereas the fault severity estimation network receives ZSC, SCR, and the anomaly score as inputs and generates a fault indicator, which directly indicates the fault severity. Several experiments are conducted to verify the reliability of the proposed method, even at various operating points and fault severities. The major advantages of this study over previous methods are as follows.

1. The method could diagnose an early-stage fault in a cable by estimating the soft fault severity before the fault become permanent state; that is, a hard fault.
2. In contrast with reference signal-based methods, in this method, the need to design a reference signal and consider physical cable parameters is eliminated because only three-phase currents are required to conduct diagnosis.
3. The method is reliable under various operating conditions that may result from irregular machine operation as well as various fault conditions that range from mild to severe.

The remainder of this paper is organized as follows. In Section 2, a soft fault and its effect on cables is described. In Section 3, the novelty detection and severity estimation method is proposed. In Section 4, the proposed method is demonstrated based on various experimental scenarios. Finally, Section 5 summarizes the study.

## 2. Soft Fault in Cable

**Figure 1:**
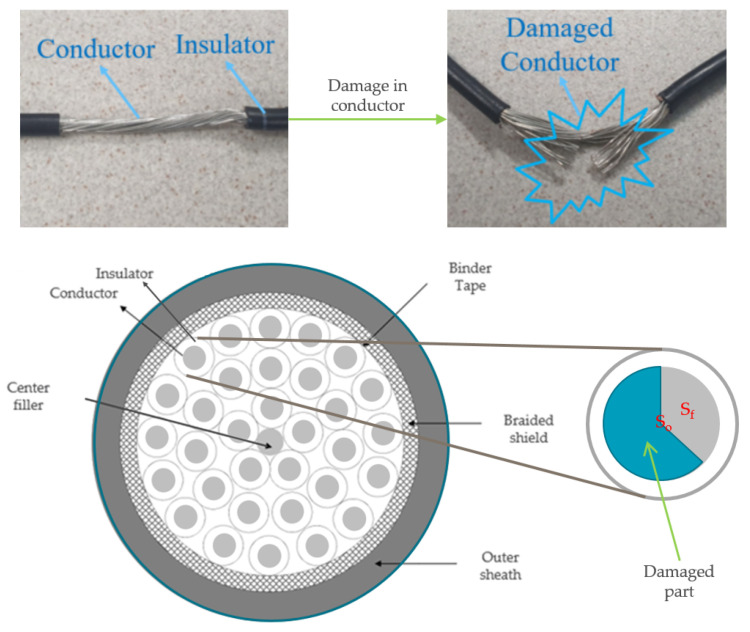
A cross-sectional view of robot control cable. A wire is composed of a conductor and an insulation layer. Damage can be induced to the wire due to harsh environment, resulting in machinery failure.

Soft faults occur because of sequential stress from harsh environments. Local modifications can be made to parts of cables, such as conductors, insulators, and shields [6]. Among them, modifications or damage to the conductor can directly affect the health state of the cable. A damaged cable is presented in Figure 1. The cross-sectional area of the conductor is reduced because of the damage, as shown in the figure. Here, $S_{o}$ denotes the cross-sectional area of a healthy conductor, and $S_{f}$ denotes the cross-sectional area of a damaged conductor.

**Figure 2:**
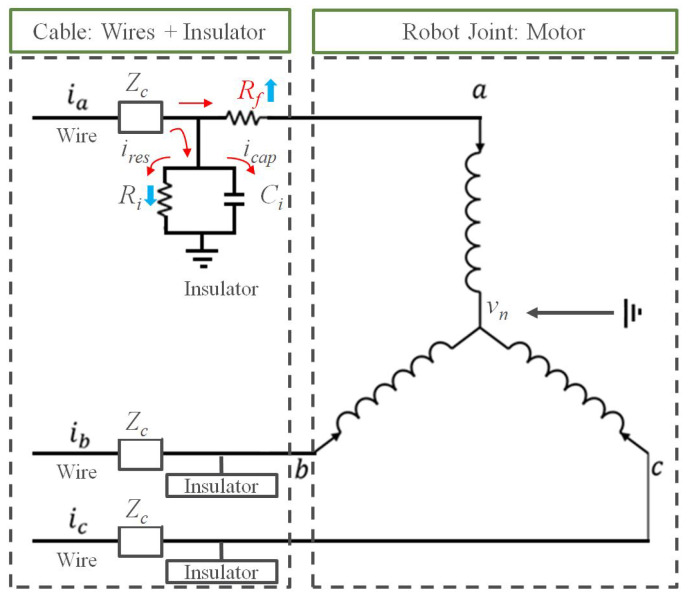
Equivalent circuit model: a three-phase control cable with a soft fault and a motor of the robot joint. A soft fault is induced in phase *a*. $Z_{c}$ is the characteristic impedance of the wire. $C_{i}$ and $R_{i}$ in parallel represent the insulator. $i_{res}$ and $i_{cap}$ are the leakage current and the current flowing through the capacitance of the insulator $C_{i}$, respectively. $V_{n}$ is the neutral voltage of the motor.

To analyze the soft faults in the cable, an equivalent circuit model is presented in Figure 2. It is assumed that a soft fault occurs in phase *a* wire. As shown in figure, the soft fault is represented as an added resistance to the phase *a* wire. The resistance $R_{f}$ denotes the induced damage in the conductor because of the fact that $R_{f}$ and $S_{f}$ are inversely correlated. Further, $R_{f}$, $S_{f}$, and their relation to the normal condition can be expressed as follows:

(1) $$\frac{S_{f}}{S_{o}}=\alpha$$

(2) $$R_{o}=\rho \frac{l}{S_{o}}$$

(3) $$R_{f}=\rho \frac{l}{S_{f}}$$

(4) $$R_{f}=\rho \frac{l}{\alpha S_{o}}=\frac{1}{\alpha }R_{o}$$

where $S_{o}$ is the normal cross-sectional area of the healthy conductor. $R_{o}$ denotes the resistance of the healthy conductor and ρ is the resistivity of the conducting material. The length of the conductor is denoted by *l*. α indicates the ratio of $S_{f}$ to $S_{o}$. The reduced area $S_{f}$ can result in a phase imbalance in the current magnitude and negative effects, including heating and energy decrease in the system [21]. Further, an imbalance in the phase impedance could result in asymmetry of the three-phase system. For healthy and soft faulty cables, the value of α is one and less than one, respectively. Mainly soft faults are discussed in this study; therefore, hard fault cases including α=0 (open-circuit) and α>1 (short-circuit) were not addressed in the proposed method. According to the relation in (4), α directly provides information on the state of the conductor. Therefore, the fault indicator (FI) can be defined as

(5) FI=1−α

In Figure 2, $Z_{c}$ is the characteristic impedance of normal wire. $C_{i}$ and $R_{i}$ in parallel denote the insulator, because the insulation layer can be represented by its capacitive and conductive characteristics [22]. $i_{res}$ is the leakage current, which is typically smaller than $i_{cap}$ in healthy wires. In case of the presence of faults in a wire, $R_{f}$ increases and $R_{i}$ decreases because the conductor and insulator are damaged. Consequently, $i_{res}$ increases due to the changed values of $R_{f}$ and $R_{i}$ according to the fault. In this case, the vector sum of the three-phase currents ($i_{a}$, $i_{b}$, $i_{c}$—three sine waves separated by 120 degrees) will have a nonzero value owing to the leakage current. Therefore, the sum—that is, the ZSC—can be used as an input for the proposed network:

(6) $$i_{a}\left(t\right)=I_{ma}cos\left(\omega t+\phi_{a}\right)$$

(7) $$i_{b}\left(t\right)=I_{mb}cos\left(\omega t+\phi_{b}-120^{\circ }\right)$$

(8) $$i_{c}\left(t\right)=I_{mc}cos\left(\omega t+\phi_{c}+120^{\circ }\right),$$

(9) $$i_{ZS}=i_{a}+i_{b}+i_{c}$$

where $i_{ZS}$ is the ZSC of the input currents. The amplitudes of each current are represented as $I_{ma}$, $I_{mb}$, and $I_{mc}$. Its corresponding phase angles are represented as $\phi_{a}$, $\phi_{b}$, and $\phi_{c}$. The magnitude of $i_{ZS}$ is approximately zero or stable for healthy wires, whereas it fluctuates significantly when there is asymmetry in the phase wires. In case of a severe fault, the asymmetry worsens. Moreover, the magnitudes of the current of each phase differ from those of the normal condition because of the asymmetry in the three-phase system. Consequently, the ratio of each phase current to the total current immediately indicates an asymmetry in the system, as follows:

(10) $$\mathrm{SSC}=\sum_{j}i_{j}^{2}$$

(11) $${\mathrm{SCR}}_{j}=i_{j}^{2}/\mathrm{SSC}$$

where *j* denotes the phase *a*, *b*, and *c*. Each phase’s SCR is defined as each squared phase current divided by the sum of squared phase currents (SSC) from all the phases. The SCR can be used for a comparison of the phase current with the total current. Therefore, it reflects a faulty three-phase network with $R_{f}$ and can be used as an input for the proposed network.

**Figure 3:**
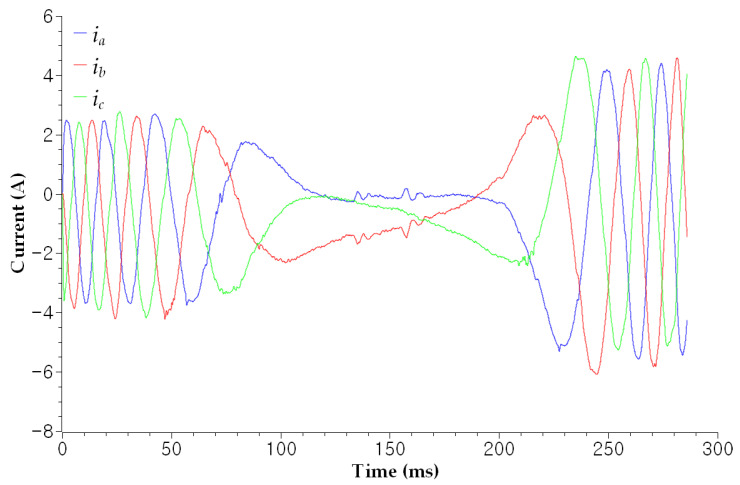
The plotted line represents the normal variation in instantaneous currents when the robot is moving. Speed, rotational direction, and amplitude varied according to the motion.

**Figure 4:**
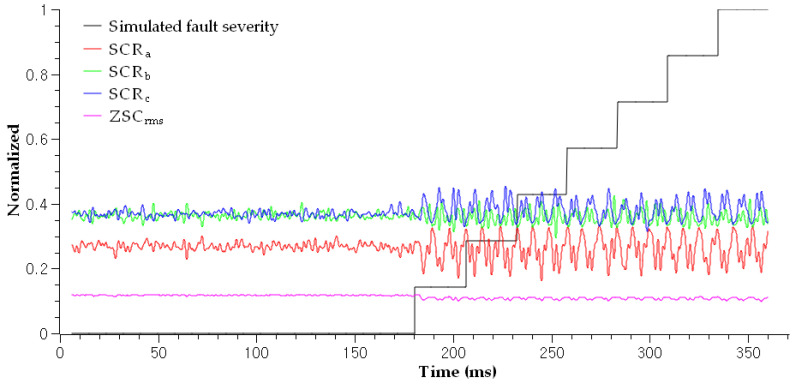
The calculated SCRs and ZSC under varying robot-moving conditions and fault conditions. SCRs and ZSC started fluctuating when faults were induced to the target cable. ZSC was root-mean-squared prior to being input to the algorithm.

The utilization of current signals originating from the operating machine with irregular motion is challenging because the amplitude, frequency, and phase sequence vary according to motion planning, as shown in Figure 3. For instance, an industrial robot installed in the production process can move its arm to perform the given motion commands, which allows the robot joint to rotate in the forward direction after rotating in the reverse direction. Even in this case, SCR and ZSC are not affected by the varying operating conditions because no phase rotation operator is involved, as shown in Figure 4. In the figure, SCRs and ZSC are stable when cable is healthy with the robot moving as mentioned above. Fluctuations appeared when varying degrees of fault were induced to the robot control cable. Still, we could not estimate the fault severity to diagnose a soft fault. The proposed diagnosis system in this paper will calculate an anomaly score of each SCR and ZSC and estimate fault severity to provide a guide for a proper maintenance plan. The analysis thus far assures that soft faults can be directly diagnosed by utilizing the ZSC and SCR.

## 3. Soft Fault Diagnosis System Structure

**Figure 5:**
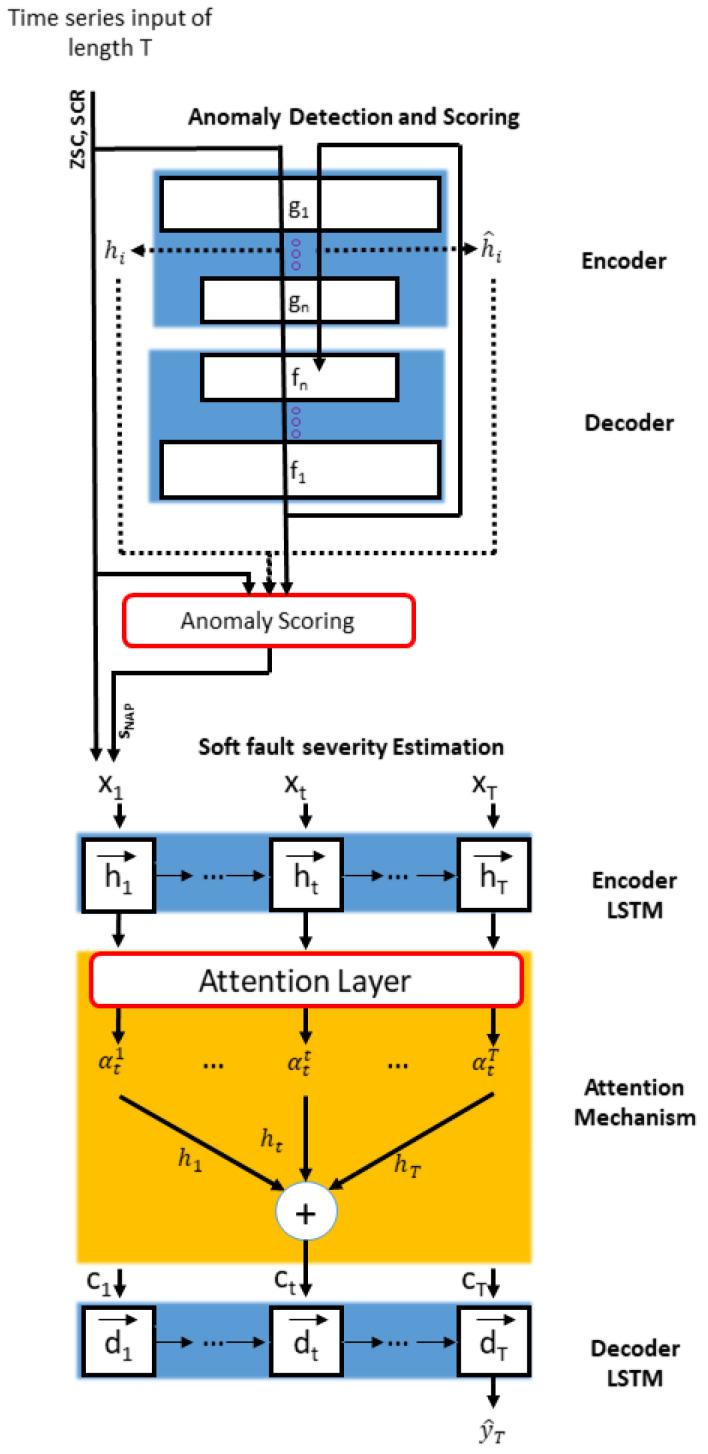
Structure of the proposed network comprising anomaly detection and fault severity estimation. The last LSTM unit of the decoder outputs is ${\hat{y}}_{T}$, which is the fault indicator.

RNNs can effectively utilize the time series data because they have the ability to consider the time-dependent information of the time series input. In this section, we propose an attention-based RNN with an autoencoder network. The structure of the proposed network comprises two parts: anomaly detection and severity estimation. In the anomaly detection network, the encoder reduces the dimensions of the input data to generate latent code, and the decoder reconstructs the input as an output to compare the input and output, whereas, in the severity estimation network, an LSTM-based RNN structure was adopted to encode the input into the feature vector and decode the estimated FI as an output. An AM was integrated into the decoder to automatically select the related hidden states through all time steps. Figure 5 presents the graphical presentation of the proposed algorithm.

### 3.1. Anomaly Scoring with Autoencoder

An autoencoder can be constructed using an encoder and a decoder. The input dimension is reduced by the encoder, whereas the decoder reconstructs the input by using the learned latent code. Therefore, A=f∘g can be considered for an autoencoder *A*, where *f* denotes the decoder and *g* denotes the encoder. Let *n* be the number of hidden layers in *f* and *g* where $g=g_{n}\circ \cdots \circ g_{2}g_{1}$ and $f=f_{1}f_{2}\circ \cdots \circ f_{n}$. The partial computations of *f* and *g* for 1≤i≤n can be

(12) $$g_{:i}=g_{i}\circ \cdots \circ g_{1}$$

(13) $$f_{n:i}=f_{i}\circ \cdots \circ f_{n}$$

The conventional anomaly scoring method compares the input and reconstructed data to detect a novel sample. Recently, it has been shown that comparing hidden spaces can generate more accurate anomaly scores [23,24]. This approach also compares the activations in the hidden space with the corresponding reconstructions in that hidden space to calculate the anomaly score. However, a hidden reconstruction cannot be directly computed. Instead, the hidden activations of the reconstructed input $g_{:i}\left(A\left(x\right)\right)$ can be used because it has been proven that hidden activations of the reconstructed input $g_{:i}\left(A\left(x\right)\right)$ are equivalent to the corresponding hidden reconstructions of the original input $\left(f_{n:i+1}\circ g\right)\left(x\right)$ [23]. This implies that hidden reconstruction values can be obtained by simply projecting the reconstructed input A(x) into the hidden spaces. If the input x and its reconstructed input A(x) are provided, their hidden activations are computed as

(14) $$h_{i}\left(x\right)=g_{:i}\left(x\right)$$

(15) $${\hat{h}}_{i}\left(x\right)=g_{:i}\left(\hat{x}\right)=g_{:i}\left(A\left(x\right)\right)$$

To obtain the anomaly score, the normalized aggregation along the pathway ($s_{NAP}$) method was adopted in this study as follows:

(16) $$s_{NAP}\left(x\right)={∥{\left(d\left(x\right)-\mu_{X}\right)}^{T}V\Sigma^{-1}-1∥}_{2}^{2}$$

where a column vector $d\left(x\right)=h\left(x\right)-\hat{h}\left(x\right)$, $h\left(x\right)=concat[h_{0}\left(x\right),\ldots ,h_{n}\left(x\right)]$, and $\hat{h}\left(x\right)=concat\left[{\hat{h}}_{0}\left(x\right);\ldots ;{\hat{h}}_{n}\left(x\right)\right]$; $h_{0}\left(x\right)=x$ and ${\hat{h}}_{0}\left(x\right)=\hat{x}$. X denotes the given training set, D denotes a matrix wherein the ith row matches $d(x_{i})$ for $x_{i}\in X$, and $\bar{D}=U\Sigma V^{T}$ must be computed for normalization, where $\bar{D}$ denotes the column-wise centered matrix of D. $\mu_{x}$ denotes the column-wise mean of *D*. Consequently, this scoring method allows the evaluation of anomalies in the current signal when a soft fault exists.

### 3.2. Fault Severity Estimation with Attention Mechanism

The fault severity estimation part comprises encoder and decoder LSTMs with an AM. The encoder generates a hidden state $h_{t}$ that summarizes the information up to the current time step t. This can be represented as follows:

(17) $$h_{t}=f\left(h_{t-1},x_{t}\right)$$

where $h_{t-1}$ is the output of the previous LSTM unit, $x_{t}$ is the current input, and *f* denotes the activation function, which is the LSTM [25]. LSTM can handle the time features of time-series data. Further, LSTM has memory cells that store, retrieve, and maintain information. It comprises the input gate $i_{t}$, output gate $o_{t}$, and forgot gate $f_{t}$. The abandoned information is controlled by the forgot gate $f_{t}$ as follows:

(18) $$f_{t}=\sigma \left(W_{f}concat\left[h_{t-1},x_{t}\right]+b_{f}\right)$$

where σ is a sigmoid function and $W_{f}$ and $b_{f}$ are the weight and bias parameters, respectively. The updating information is controlled by the input gate $i_{t}$, which can be represented by

(19) $$i_{t}=\sigma \left(W_{i}concat\left[h_{t-1},x_{t}\right]+b_{i}\right)$$

where $W_{i}$ is the weight parameter of the input gate and $b_{i}$ is the offset term. Further, the long-term state is denoted by $s_{t}$ and $o_{t}$ is the output gate. These can be expressed as follows:.

(20) $$o_{t}=\sigma \left(W_{o}concat\left[h_{t-1},x_{t}\right]+b_{o}\right)$$

(21) $$s_{t}=f_{t}⊙s_{t-1}+i_{t}⊙\tanh\left(W_{s}concat\left[h_{t-1},x_{t}\right]+b_{s}\right)$$

where $W_{o}$ is the weight parameter of the output gate and $b_{o}$ is the offset term. Further, $W_{s}$ is the weight parameter of the state and $b_{s}$ is the bias parameter. The output $h_{t}$ of the LSTM cell can be represented as

(22) $$h_{t}=o_{t}⊙\tanh\left(s_{t}\right)$$

where ⊙ denotes the Hadamard product. Hidden states $h_{t}$ are created across all time steps to summarize time-dependent information.

The encoded input information was decoded by the decoder network to generate the estimated output ${\hat{y}}_{T}$. LSTM is integrated into the decoder to estimate the output from time dependencies. Moreover, an AM is also integrated into the network to automatically select the important encoded hidden states through time steps. AM strengthens the influence of important and relevant information by focusing on output variables with a significant impact and ignoring uninterested ones. The attention weight can be computed as follows:

(23) $$e_{t}^{k}=v_{d}^{T}\tanh\left(W_{d}concat\left[d_{t-1},s_{t-1}^{\prime }\right]+U_{d}h_{k}\right)$$

(24) $$\alpha_{t}^{k}=\frac{\exp(e_{t}^{k})}{\sum_{i=1}^{T}\exp\left(e_{t}^{i}\right)},1\leq k\leq T$$

where $v_{d}$ and $W_{d}$ are the weighting coefficients and $U_{d}$ denotes the bias coefficient. The attention weights $\alpha_{t}^{k}$, which are obtained by normalizing the weights $e_{t}^{k}$ using the softmax function, represent the importance of each hidden state from the network. The context vector $c_{t}$ can be denoted by

(25) $$c_{t}=\sum_{i=1}^{T}\alpha_{t}^{i}h_{i}$$

This is provided to the decoder as input, and the hidden state of the decoder can be written as

(26) $$d_{t}=f\left(d_{t-1},c_{t-1}\right)$$

$d_{t}$ denotes the decoder hidden state at time step *t*, and *f* is a nonlinear activation function, which is the LSTM. The hidden state $d_{t}$ can be updated as explained previously in the encoder. Thus, the fault severity output ${\hat{y}}_{T}$ can be written as

(27) $${\hat{y}}_{T}=F\left(x_{1},x_{2},\cdots ,x_{T}\right)=v_{y}^{T}\left(W_{y}concat\left[d_{T},c_{T}\right]+b_{y}\right)+b_{v}$$

where *F* denotes the approximate function for estimating fault severity from the given input vectors X=$\left(x_{1},x_{2},\cdots ,x_{T}\right)$, and $W_{y}$ and $b_{y}$ are the weight matrix and bias terms, respectively. Consequently, the fault severity can be estimated by a linear function, where $v_{y}^{T}$ and $b_{v}$ are the weight and bias, respectively.

### 3.3. Training Procedure

A novelty detection and fault severity estimation network was constructed using the Tensoflow framework [26]. The Adam optimizer [27] was used to train the model, and the sizes of the mini-batches were 128 and 20 for the novelty detection and fault severity estimation networks, respectively. Further, the sizes of the hidden states were 30 and 128 for the novelty detection and fault severity estimation networks, respectively. The time length *T* of the time-series input was 12. In addition, the $l_{2}$ loss function was used for the standard back propagation, and the objective functions for the two networks are as follows:

(28) $$l_{2}\left(x,\hat{x}\right)=\frac{1}{N}\sum_{i=1}^{N}{\left({\hat{x}}_{i}-x_{i}\right)}^{2}$$

(29) $$l_{2}\left(y_{T},{\hat{y}}_{T}\right)=\frac{1}{M}\sum_{j=1}^{M}{\left({\hat{y}}_{T}^{j}-y_{T}^{j}\right)}^{2}$$

### 3.4. Structure of Diagnosis System

**Figure 6:**
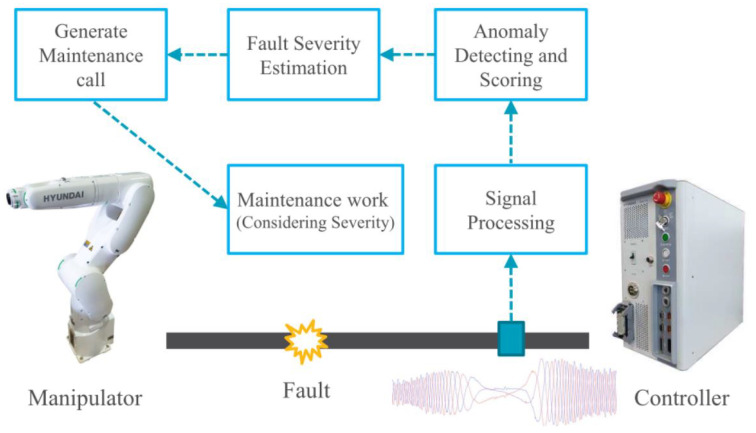
The industrial robot system with the proposed diagnosis method. It comprises signal processing, anomaly detection, and fault severity estimation components. If the detected fault severity exceeds the threshold limit, a maintenance call is generated for the staff.

The structure of the diagnostic system is shown in Figure 6. It comprises signal processing, anomaly assessing, and fault severity estimation components. The data sample from the time-series input comprises SCR, ZSC, and $s_{NAP}$, where the size of the input sample was a window of 9000 time steps. When computing SCR, ZSC, and $s_{NAP}$ using (11), (9), and (16), respectively, the raw input data were passed through a low-pass filter to remove unnecessary frequency components. The calculated fault indicator was used as the label of the network.

## 4. Experimental Results

### 4.1. Experimental Setup

**Figure 7:**
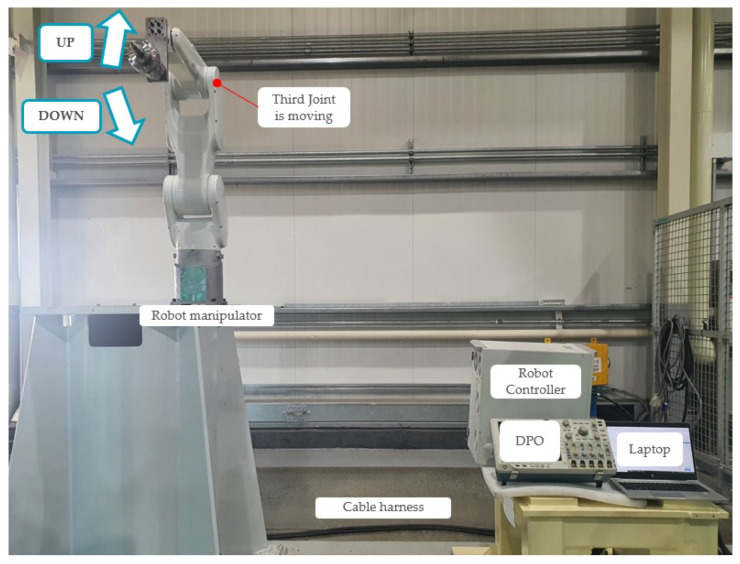
Experimental setup: The industrial robot test rig with the cable harness and the controller and measure kit. The manipulator moves up and down to vary the operating conditions.

The experimental setup is illustrated in Figure 7. A HYUNDAI HH7 robot manipulator and its controller were installed to demonstrate the proposed method. The cable harness comprised control and signal cables, with the control cable targeted for conducting the fault diagnosis. The detailed characteristics of the target cable are represented in Table 1. The cable consisted of 32 wires, each of which consisted of an insulator and conductor. Further, each conductor was composed of 30 flexible strands, each measuring 0.25 mm in diameter. Further, a Tektronix DPO 4104 B phosphor oscilloscope was used to measure current signals at a sampling rate of 100 kHz. We simulated various faults and operating conditions in the third joint of the robot manipulator.

### 4.2. Experimental Scenarios

To simulate the fault conditions, soft faults were created manually in the wire of phase *a*, as shown in Figure 1. Artificial faults exhibit a range of fault severities, from mild to severe. The fault severity and corresponding scenarios are listed in Table 2. The datasets according to each fault severity were acquired as follows. First, a normal cable was damaged repeatedly according to the damage cases. There are eight cases according to the fault severity in the table. For example, 28 cutting is the most severe fault case, whose α value is small, and 93.4% of $S_{o}$ is damaged. However, the four cuts are mild faults, whose α value is large, and 13.4% of $S_{o}$ is damaged. Moreover, the experiments were conducted under actual operating conditions wherein the manipulator moved up and down according to the motion commands, as shown in Figure 7. In the figure, the robot joint rotated inversely following forward rotation. Consequently, the speed, rotational direction, and amplitude of the three-phase currents varied according to the robot movement, as shown in Figure 3. As mentioned previously, eight scenarios and acquired datasets were prepared to conduct experiments under these varying conditions.

### 4.3. Result and Analysis

**Figure 8:**
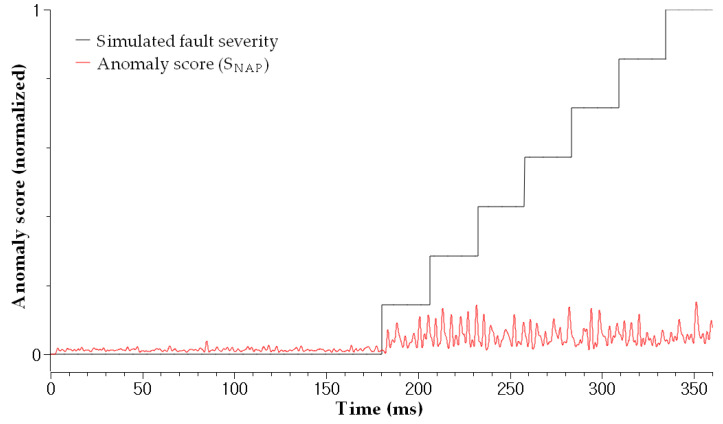
The simulated soft fault severity and the calculated anomaly score for each fault severity. From this anomaly score, we could detect presence of a fault.

**Figure 9:**
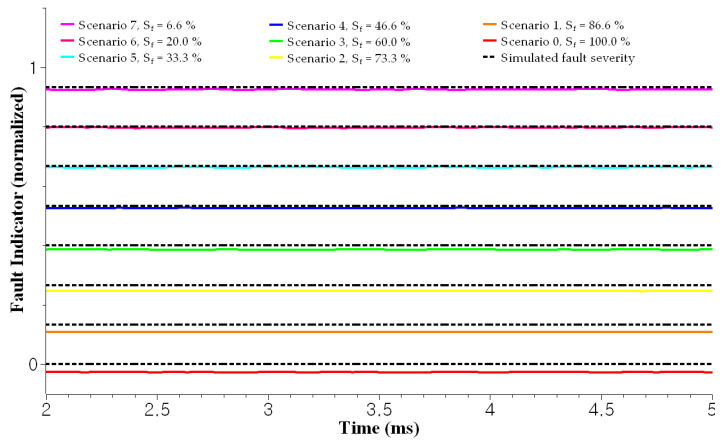
The estimated fault severity for each scenario: $S_{f}$ = 100.0%∼6.6% corresponds to scenario 0∼scenario 7, respectively. From this estimated severity, we could diagnose health state of the target cable.

The test results for the scenarios demonstrate that the proposed method can effectively diagnose soft faults. First, the autoencoder successfully detected the abnormal state and calculated anomaly scores, as shown in Figure 8. The results confirmed the suitability of the time-series input—that is, ZSC and SCR—for soft fault diagnosis by reflecting the power network imbalance. Second, the fault severities of the induced damages were correctly estimated by the attention-based RNN architecture, as shown in Figure 9. The result confirms that the RNN inputs—that is, ZSC, SCR, and $s_{NAP}$—are appropriate for estimating the fault severity of a particular fault. As shown in Figure 9 ($S_{f}$ = 100.0%), the FI for healthy cable was approximately zero for varying operating conditions, such as speed, amplitude, and phase. The FI increased as the severity increased according to Scenario 7, as shown in Figure 9 ($S_{f}$ = 6.6%). Further, the faulty cable and healthy cable were clearly distinguished by the FI. Thus, it can be concluded that the FI is independent of the varying operating conditions resulting from the robot manipulator movements. Therefore, the proposed method is applicable to practical online diagnosis when automated machines operate in smart factories. Figure 9 ($S_{f}$ = 86.6%) shows the test result for Scenario 1. The fault condition was the least severe compared to the other scenarios, implying that diagnosing Scenario 1 was more difficult compared to other scenarios. Four strands of the 30 strands were damaged artificially to simulate very early soft fault conditions, where only 13.4% of the cross-sectional area of the conductor was damaged. In this case (early case even among the soft fault cases), the automated machines can work without any fault symptoms. Compared to the magnitude observed in Figure 9 ($S_{f}$ = 6.6%), the magnitude of the corresponding FI in Figure 9 ($S_{f}$ = 86.6%) was much smaller. Nevertheless, the early stages of soft faults can be diagnosed using the estimated FI, even under mild fault conditions. The FI estimation results for all scenarios are shown in Figure 9 ($S_{f}$ = 6.6%∼100.0%). The estimated FI tended to be more accurate for severe fault cases (Scenarios 4 to 7) because the magnitude of the simulated FI was much smaller in less severe scenarios (Scenario 1 to 3). Table 3 confirms this trend by showing mean square errors (MSE) of each scenario. Consequently, we can assert that the proposed method achieves a soft diagnosis capability under various fault and operating conditions, rendering this method applicable to practical problems.

### 4.4. Comparison with Other Studies

The proposed method was compared to other methods by considering several aspects, as listed in Table 4. Reflectometry-based methods [28,29,30,31] require additional equipment, such as arbitrary waveform generators, voltage sensors, and signal-coupling devices, to conduct the diagnosis. Thus, an extra budget is required to purchase the equipment. Space and accessibility for installing the equipment are also required. Furthermore, expert and cable parameters are required to analyze the returned reference signal, which must also be designed before conducting a diagnosis for each target cable. Moreover, only sstdr [30] among other reflectometry-based methods is capable of diagnosing cable faults in online conditions. The study by [9] overcame this problem by suggesting a method that is applicable to both online and offline diagnosis using a reference burst signal; however, it still requires a signal generator and a signal-coupling device. Several studies have utilized current signals when diagnosing cable faults [10,11], which are preferred over other methods because they do not require extra equipment. However, the study by [10] could not handle the varying conditions wherein the amplitude and phase angles varied according to the movements of automated machines. The study by [11] overcame this problem by adopting a novelty detection approach, even under varying operating conditions. However, the diagnostic purpose of this study was fast fault detection and isolation when an early-stage fault occurs in the cable; thus, severity estimation needs to be supported. The severity estimation capability is important for maintenance staff because they can assess the fault condition and consequently build a proper maintenance plan, such as cable replacement and overhaul. Thus, the fault severity estimation capability of the proposed method is the most significant difference compared to previous methods. Consequently, the comparison results show that the proposed method is an appropriate approach for addressing cable fault problems in industries.

## 5. Conclusions

This study proposed a new diagnostic approach for cable faults, which is particularly applicable in the field of industrial automation. The diagnosis method comprised a novelty detection and severity estimation network, wherein an attention-based RNN was integrated into the latter network to consider time-dependent information and overcome the long-term dependence problem. RNN is a type of neural network which utilizes sequential data. In this paper, RNN was adopted because the time series data obtained from the cable are sequential and time dependent. The inputs for the novelty detection network were ZSC and SCR, and an anomaly score was generated. The severity estimation network accepted the anomaly score and estimated the fault indicator, which directly represents fault severity. The proposed method is cost effective and saves space because it does not require additional equipment to conduct the diagnosis. Moreover, cable parameters and expert knowledge are not required. Furthermore, the proposed method can provide maintenance staff with a fault severity estimation capability to assess the fault situation. Experiments on an industrial application proved that the new approach works effectively and accurately under various faults and operating conditions. Future studies will focus on intermittent faults in flexible cables for moving applications using an explainable AI approach with a data fusion algorithm.

## Figures and Tables

**Table 1:** Specifications of the control cable.

Subjects	Unit	Specification
Total number of wires in cable	-	32
Cross-sectional area of a conductor	mm${}^{2}$	1.5
Number of strands of a conductor	-	30
Cross-sectional are of a strand	mm${}^{2}$	0.25
Maximum resistance of a wire	Ω/km	17.7
Material of insulation layer	-	PVC
Thickness of insulation layer	-	0.36
Shield type	-	Braided
Outer diameter	mm	20.4

**Table 2:** Experimental scenarios.

Scenarios	Damage Cases	FI	$S_{f}$ in %	Number of Dataset
0 (Normal)	0 cut	0.000	100.0	85,000
1	4 cut	0.134	86.6	85,000
2	8 cut	0.267	73.3	85,000
3	12 cut	0.400	60.0	85,000
4	16 cut	0.534	46.6	85,000
5	20 cut	0.667	33.3	85,000
6	24 cut	0.800	20.0	85,000
7	28 cut	0.934	6.6	85,000

**Table 3:** MSE of each scenario.

Scenarios	Damage Cases	FI	MSE
0 (Normal)	0 cut	0.000	72.31 × $10^{-5}$
1	4 cut	0.134	65.66 × $10^{-5}$
2	8 cut	0.267	42.29 × $10^{-5}$
3	12 cut	0.400	19.29 × $10^{-5}$
4	16 cut	0.534	7.17 × $10^{-5}$
5	20 cut	0.667	1.80 × $10^{-5}$
6	24 cut	0.800	0.98 × $10^{-5}$
7	28 cut	0.934	8.85 × $10^{-5}$

**Table 4:** Comparison with other studies.

	Utilizing Current Signal [Proposed Method] [10,11]			Utilizing Injected Reference Signal [9,28,29,30,31]				
Estimation of soft fault severity	O	X	X	X	X	X	X	O
Online diagnosis under varying conditions	O	X	O	O	X	X	O	X
Require reference signal design	X	X	X	O	O	O	O	O
Require domain knowledge and cable parameters	X	X	X	X	O	O	O	O
Require waveform generator	X	X	X	O	O	O	O	O

For the first two rows, O is better. For the last three rows, X is better.

## Data Availability

The data presented in this study are available on request from the corresponding author. The data are not publicly available because it is company confidential information.
